# Individual and Contextual Characteristics Associated With Alcohol Use Among Brazilian Adolescents

**DOI:** 10.3389/ijph.2022.1604397

**Published:** 2022-06-02

**Authors:** Patrycia Sarah Martins Arruda, Aline Natália Silva, Ana Elisa Madalena Rinaldi, Luciana Saraiva da Silva, Catarina Machado Azeredo

**Affiliations:** School of Medicine, Federal University of Uberlândia, Uberlândia, Brazil

**Keywords:** health education, adolescent behavior, adolescent, alcoholic drinks, educational institutions

## Abstract

**Objective:** To analyze the association between individual and contextual characteristics with alcohol indicators of experimentation, use in the last 30 days and drunkenness in Brazilian adolescents.

**Methods:** Cross-sectional study based on data from 100,914 student attending 9^th^ grade from the 2015 National School Health Survey. Multilevel logistic regression models were performed for the outcomes: alcohol experimentation; use in the last 30 days and drunkenness; and exposures, adjusted for adolescents’ sociodemographic characteristics.

**Results:** Girls were more likely to experiment alcohol (OR = 1.09; 95% CI = 1.05–1.12), use it in the last 30 days (OR = 1.09; 95% CI = 1.00–1.13) and less prone to drunkenness (OR = 0.90; 95% CI = 0.87–0.93). A higher proportion of public-school students reported drinking. There was a positive association of substance use by parents, peers and the adolescents themselves with the outcomes. Having a policy of banning alcohol consumption at school was associated with a greater chance of alcohol experimentation in public schools.

**Conclusion:** Exposure to legal and illegal substances by friends, family members and a prohibitive school environment favored the outcomes.

## Introduction

Alcohol is the most prevalent psychoactive substance use among adolescents in the world [1]. Worldwide, 43% of adolescents over the age of 15 reported having consumed alcoholic beverages in the last year [2]. In Brazil, 66.6% of students from the 6th to the 9th grade had already tried alcohol and 21.8% had already been drunk in 2012 [3].

Alcohol consumption is determined by adolescents individual and behavioral characteristics and their social context, including the family environment, friends and the school [4]. Living with peers, parents or older family members who use alcohol is associated with a greater chance of adolescents also using and maintaining this behavior throughout life [3, 5–10]. In Porto Velho, Brazil, alcohol experimentation at home was reported by 39.2% of students, most of them aged between 12 and 13 years [9]. They also reported the habit of drinking primarily with friends and family members [9].

In addition to individual and relational characteristics, contextual factors, including the school environment, are associated with adolescent alcohol consumption [10–12]. The school is an important space for the development of policies aimed at reducing the consumption of alcohol and other substances such as illicit drugs and cigarettes [13, 14]. Policies in well-established educational institutions have positive results in student behaviors, such as reducing alcohol consumption, improving academic performance and decreasing problematic attitudes [15, 16].

After the adoption of prevention policies at school, a study conducted in the United States showed a 22% reduction in alcohol consumption in the last 30 days and a 25% reduction in alcohol-related consequences among adolescents [17], while an European study showed a 22% reduction in alcohol-related behavioral problems. In a study carried out in southern Brazil, there was no association between the absence of specific preventive actions for alcohol use and the promotion of healthy habits and alcohol consumption among schoolchildren [18]. Onrust et al. [19] emphasize in their meta-analysis that there is no consistency in the results on the effectiveness of school programs for the prevention of substance use.

Although the school offers resources to intervene in health and in the adoption of preventive measures in adolescent substance consumption [3, 13, 20] studies that conducted a detailed analysis on the subject are scarce in Brazil [9, 18]. In addition, experimentation of alcoholic drinks, regular use and excessive consumption leading to drunkenness may have different determinants [21]. Therefore, investigations on the determinants of different indicators of alcohol use are extremely important in the formulation of specific policies and actions. Thus, this study was designed to analyze the association between individual and contextual characteristics with alcohol indicators of experimentation, use in the last 30 days and episode of drunkenness in Brazilian adolescents.

## Methods

### Population, Sampling and Data Collection

This is a cross-sectional study based on data from the 2015 National School Health Survey (PeNSE) [22, 23]. PeNSE was carried out through a partnership between the Ministry of Health, the Ministry of Education and the Brazilian Institute of Geography and Statistics (IBGE). The sample design of PeNSE 2015 [22, 23] ensured representativeness for the whole country and for the five major geographic regions. We used data from sample 1 of PeNSE [22, 23], with adolescents enrolled in public and private schools, who attended the 9th grade of elementary school, from the 26 capitals of the Brazilian states and the Federal District in 2015. Schools were the primary sampling units and classes the secondary sample units. The probability of selecting the school was proportional to its size, defined by the number of existing 9th grade classes. One class was selected from each school that reported having up to two 9th grade classes, and two classes from each school that reported having three or more 9th grade classes. In addition, all students in the chosen classes were invited to participate in the survey.

Two questionnaires were used. The first intended for students in a self-administered manner, using smartphones, based on the questionnaire of the Global School-based Student Health Survey (GSHS), containing the following themes: socioeconomic aspect; family context; and substance use (tobacco; alcohol and illicit drugs). The second questionnaire was administered to the principals or those responsible for the school and completed by the IBGE interviewer, containing questions about the school’s structure, physical dimension, spaces, equipment, practices and routines, policies adopted by the institution and situations around the school. More information about the Survey is available in Oliveira et al. [23].

### Description of the Variables of Interest

#### Outcomes

The adolescents answered about the use of alcoholic drinks in their lives (no/yes), hereinafter called alcohol experimentation; about alcohol use in the last 30 days (no/yes); and episode of drunkenness in life (no/yes).

The questions were as follows:

Alcohol experimentation: “Have you ever had a drink of alcohol?” (one dose is equivalent to a can of beer or a glass of wine or a dose of cachaça or whiskey etc.), with the coding “No” and “Yes”; Alcohol use in the last 30 days: “In the last 30 days, how many days have you had at least one glass or one dose of alcohol?” (one dose is equivalent to a can of beer or a glass of wine or a dose of cachaça or whiskey etc.), with the coding “No” (no day in the last 30 days) and “Yes” (1 or more days); Episode of drunkenness: “In your life, how many times have you drank so much that you were really drunk?,” with the coding “No” (never) and “Yes” (1 or more times).

#### Exposure Variables

The exposure variables were subdivided into individual and contextual variables. The following individual variables were assessed: having friends (none; 1 or 2; 3 or more friends); having friends who consume alcoholic drinks (no; a few, most, do not know); having friends who use illicit drugs (no; a few, most, do not know); having smoking parents (no; one smokes, both smokes, do not know). The illicit drugs considered were marijuana, cocaine, crack, cola, loló, perfume launcher, ecstasy, oxy, etc.

The following contextual variables were assessed the administrative situation of school (public/private), the existence of health policies at the school, such as the presence of a committee coordinating health actions at the school (no/yes), the implementation of actions of the Health at School Program (PSE) in public schools (no/yes); the practice of joint actions with Primary Care (PC) (no/yes) at school; principal’s report of use of cigarettes by teachers at school (no/yes); principal’s report of use of cigarettes by students at school (no/yes), and the existence of policy banning alcohol consumption at school (no/yes).

The PSE is an intersectoral policy of the Ministry of Health and the Ministry of Education, implemented through Decree no. 6,286/2007 [14], which promotes actions of attention, prevention, promotion and assistance in health, including, among others, the prevention and reduction of alcohol consumption in public schools. The presence of the committee that coordinates health actions at school and the practice of joint actions with PC collaborate to promote integral health and education, with a view to addressing the vulnerabilities that compromise the full development of children and young people.

#### Covariables

The following covariables were considered: sex (female; male); age (categorized as: ≤ 13; 14 or 15; 16 years or over); color or race (white; black; Asian; brown; and Native Brazilian Indian); maternal education (incomplete elementary school; complete elementary school; complete high school; complete higher education). In addition to these variables, information on cigarette consumption by the adolescent at any time in life (no/yes) was included, as well as cigarette consumption in the last 30 days (no/yes), consumption of illicit drugs at least once in life (no/yes) and consumption of illicit drugs in the last 30 days (no/yes). There was no income variable in the questionnaire, so the score for goods and services was created, based on the self-reported possession of landline, cell phone, computer, internet at home, car and access to maid service. Each item was weighted by the inverse of the frequency of possession and the score for each student was obtained by adding the weighted scores, as described by Levy et al. [22]. Finally, the score was divided into terciles, the first tercile being composed of those with the lowest score and the third tercile of those with the highest score.

The categories of answers “do not know” or “missing” in covariates were preserved in the analyzes to minimize sample loss. However, in multilevel models these categories have been omitted. The response rate was 98.6%, totaling 2,979 schools and 100,914 students.

### Statistical Analysis

Initially, the variables were examined by means of descriptive analyses, being expressed in prevalence according to the type of school administration (public and private) and general prevalence for the set of schools. The association between alcohol consumption outcomes (alcohol experimentation; alcohol use in the last 30 days and episode of drunkenness) and exposure variables (individual and contextual) was analyzed using multilevel logistic regression models to obtain the odds ratio (OR) with the respective 95% confidence intervals (CI). The analyzes were performed stratified for public and private schools and for the total set of schools (crude and adjusted models). For the adjustment, the covariables (sex, age, maternal education, skin color, score of goods, information on cigarette and illicit drugs consumption by the adolescent) were used. The variable on the implementation of PSE actions was used only in the public school model, as it is a policy implemented only in those schools. Values where *p* < 0.05 were considered statistically significant. The data were analyzed using the Stata SE 14.0 software.

### Ethical Aspects

PeNSE data are publicly available on the IBGE website, without any identification of the participants. PeNSE 2015 was approved by the National Research Ethics Commission (CONEP), of the National Health Council (CNS), through CONEP opinion No. 1.006.467.

## Results

The sex distribution of the adolescents was similar, most of them were between 14 and 15 years old and self-declared brown skin color/race. The maternal education levels most reported by students from public and private schools were incomplete elementary school and complete higher education, respectively (Table 1). Students from public schools reported greater experimentation, last-30-day consumption, and episode of drunkenness than those from private schools, and most schools had policies prohibiting alcohol use. Joint actions with PC and actions coordinated by a school committee were practiced in 74.6% and 35.7% of public schools and in 34.4% and 42.2% of private schools, respectively. PSE actions were implemented in 43.1% of public schools (Table 1).

**TABLE 1:** Prevalence of individual and contextual characteristics by type of school (public, private). National School Health Survey, Brazil, 2015.

Variables	Public school % (95%CI)	Private school % (95%CI)	Total % (95% CI)
Individuals (n = 100.914)	*n* = 611	*n* = 2.368	
Sex			
Female	51.5 (50.8–52.2)	51.0 (49.5–52.4)	51.8 (51.5–52.1)
Male	48.4 (47.7–49.1)	48.9 (47.5–50.4)	48.1 (47.8–48.4)
Age (years)			
<13	16.8 (15.6–18.0)	27.1 (24.8–29.6)	16.9 (16.7–17.1)
14 or 15	71.0 (69.9–72.2)	69.3 (66.9–71.7)	71.0 (70.7–71.3)
≥16	12.1 (11.4–12.8)	3.4 (2.8–4.1)	11.9 (11.8–12.2)
Color or race			
White	33.1 (32.1–34.2)	53.8 (50.9–56.7)	33.0 (32.8–33.3)
Black	14.2 (13.6–14.8)	7.82 (6.81–8.9)	12.5 (12.3–12.7)
Asian	3.8 (3.6–4.1)	5.3 (4.8–6.0)	4.4 (4.3–4.6)
Brown	45.2 (44.2–46.1)	29.8 (27.6–32.0)	45.9 (45.6–46.2)
Indigenous	3.3 (3.0–3.5)	3.0 (2.5–3.5)	3.7 (3.6–3.8)
Maternal education			
Incomplete elementary school	27.7 (27.0–28.4)	6.5 (5.5–7.6)	23.2 (23.0–23.5)
Complete elementary education	13.2 (12.7–13.7)	8.4 (7.6–9.2)	12.0 (11.8–12.2)
Complete high school	21.5 (20.8–22.2)	28.5 (26.5–30.6)	22.9 (22.6–23.1)
Complete higher education	8.4 (7.9–8.8)	42.0 (38.8–45.2)	16.9 (16.7–17.1)
Score for goods			
1° Tertile	41.4 (40.1–42.6)	7.7 (6.5–9.0)	39.6 (39.3–39.9)
2° Tertile	32.6 (31.9–33.3)	22.9 (21.2–24.7)	29.7 (29.4–30.0)
3° Tertile	25.9 (24.7–27.1)	69.2 (66.6–71.7)	30.5 (30.2–30.8)
Experienced alcohol^a^	53.6 (52.7–54.5)	48.7 (46.8–50.6)	51.7 (51.4–52.0)
Drank alcohol in the last 30 days ^a^	24.2 (23.5–24.9)	21.2 (19.5–22.9)	22.1 (21.9–22.4)
Had an episode of drunkenness^a^	22.2 (21.4–22.9)	16.5 (15.4–17.7)	20.2 (20.0–20.5)
Experienced cigarette^a^	19.3 (18.6–19.9)	12.5 (11.4–13.7)	18.3 (18.0–18.5)
Have used cigarettes in the last 30 days	5.9 (5.5–6.3)	3.6 (3.0–4.3)	5.5 (5.1–5.4)
Experienced drugs (marijuana, cocaine, crack, cola, lolo, perfume launcher, ecstasy, oxy etc)^a^	9.3 (8.1–9.8)	6.7 (6.0–7.5)	8.4 (8.2–8.6)
Have used drugs in the last 30 days	4.2 (3.9–4.6)	3.3 (2.8–3.9)	3.8 (3.7–3.9)
Having friends			
None	4.4 (4.2–4.7)	3.0 (2.4–3.7)	4.0 (3.9–4.2)
1 or 2	19.1 (18.5–19.7)	16.9 (15.7–18.2)	19.1 (18.8–19.3)
3 or more	76.3 (75.7–76.9)	79.9 (78.6–81.2)	76.7 (76.5–77.0)
Friends use alcohol^a^			
No	19.0 (18.4–19.6)	19.6 (18.0–21.2)	20.1 (19.9–20.4)
A few	50.9 (50.3–51.6)	54.6 (52.7–56.5)	51.4 (51.1–51.7)
Most	20.7 (20.0–21.5)	18.1 (16.7–19.6)	19.7 (19.5–20.0)
Do not know	9.1 (8.7–9.5)	7.5 (6.7–8.5)	8.6 (8.4–8.7)
Friends use illicit drugs			
No	46.0 (45.0–47.1)	52.3 (50.2–54.4)	48.9 (48.6–49.2)
A few	31.5 (30.7–32.3)	31.0 (28.7–33.4)	30.0 (29.7–30.3)
Most	6.2 (5.7–6.7)	3.1 (2.6–3.7)	5.1 (5.0–5.3)
Do not know	16.1 (15.5–16.6)	13.4 (12.4–14.4)	15.8 (15.6–16.0)
Parents smoke			
No	69.9 (69.2–70.6)	81.9 (80.7–83.0)	73.8 (73.5–74.0)
One smokes	23.0 (22.4–23.7)	14.2 (13.3–15.2)	20.1 (19.0–20.4)
Both smoke	4.6 (4.3–5.0)	2.4 (2.0–3.0)	3.7 (3.6–3.8)
Do not know	2.2 (2.0–2.4)	1.3 (1.0–1.6)	2.2 (2.1–2.3)
Contextual (*n* = 2.979)			
School type			
Public	—	—	79.4 (79.1–79.6)
Private	—	—	20.5 (20.3–20.8)
Committee that coordinates health actions at school	35.7 (32.1–39.5)	42.2 (35.0–49.8)	36.1 (35.8–36.4)
PSE actions implemented^a^	43.1 (39.8–46.5)	—	—
Joint actions with PC^a^	74.6 (70.9–77.9)	34.4 (27.9–41.4)	67.1 (66.8–67.4)
Principal’s report of use of cigarettes by teachers at school^a^	15.1 (12.5–18.2)	6.6 (4.1–10.7)	12.3 (12.1–12.5)
Principal’s report of use of cigarettes by students at school^a^	20.5 (17.5–23.9)	5.3 (3.0–9.1)	20.8 (20.5–21.0)
Policy banning alcohol consumption at school^a^	90.4 (88.5–92.1)	92.4 (87.9–95.3)	90.5 (90.3–90.7)

a Presentation of answers “yes”.

CI, confidence interval.

In the model for the total sample, being female, having already tried cigarettes and drugs, having friends who used alcohol and drugs, having parents who smoke, and being a student in schools that had a policy of prohibiting the consumption of alcoholic beverages was associated with a greater probability of experimenting with alcohol. Students whose principals reported being aware that teachers smoked inside the school were more likely to try alcohol in the total model and in the model for public schools. In the stratified model for public schools, the implementation of PSE actions was associated with less alcohol experimentation in the crude model, although the association was not statistically significant after the adjustments (Table2).

**TABLE 2:** Odds ratio estimated by multilevel analysis for the association between individual and contextual characteristics and alcohol experimentation by Brazilian adolescents. National School Health Survey, Brazil, 2015.

Variables	Public schools		Private schools		Total	
	Crude model OR (95%CI)	Adjusted model OR (95%CI)	Crude model OR (95%CI)	Adjusted model OR (95%CI)	Crude model OR (95%CI)	Adjusted model OR (95%CI)
Individuals						
Sex						
Male	1	1	1	1	1	1
Female	1.09 (1.06–1.12)	1.12 (1.08–1.16)	1.04 (0.99–1.11)	0.98 (0.92–1.05)	1.08 (1.05–1.11)	1.09 (1.05–1.12)
Experienced cigarette						
No	1	1	1	1	1	1
Yes	14.38 (13.57–15.24)	7.41 (6.96–7.90)	20.59 (17.42–24.34)	8.02 (6.70–9.60)	15.02 (14.22–15.87)	7.46 (7.03–7.92)
Experienced drugs						
No	1	1	1	1	1	1
Yes	18.33 (16.52–20.33)	3.67 (3.26–4.12)	28.09 (21.47–36.74)	4.26 (3.16–5.75)	19.59 (17.78–21.58)	3.77 (3.38–4.20)
Having friends						
None	1	1	1	1	1	1
1 or 2 friends	1.15 (1.07–1.24)	1.08 (0.98–1.18)	1.12 (0.93–1.34)	1.07 (0.87–1.33)	1.14 (1.07–1.23)	1.07 (0.99–1.17)
3 or more friends	1.00 (0.93–1.07)	1.02 (0.94–1.11)	0.99 (0.84–1.17)	1.02 (0.83–1.25)	1.00 (0.97–1.11)	1.02 (0.94–1.10)
Friends use alcohol						
No	1	1	1	1	1	1
A few	4.59 (4.40–4.79)	3.24 (3.08–3.39)	4.65 (4.27–5.07)	3.26 (2.98–3.58)	4.61 (4.43–4.79)	3.23 (3.10–3.37)
Most	14.94 (14.13–15.79)	6.61 (6.20–7.05)	18.66 (16.64–20.93)	7.72 (6.78–8.79)	15.61 (14.85–16.41)	6.79 (6.41–7.19)
Friends use drugs						
No	1	1	1	1	1	1
A few	4.86 (4.68–5.04)	2.00 (1.92–2.09)	5.46 (5.08–5.88)	2.24 (2.06–2.43)	4.98 (4.82–5.15)	2.05 (1.98–2.13)
Most	9.26 (8.52–10.06)	1.52 (1.37–1.68)	16.71 (12.91–21.63)	2.18 (1.62–2.94)	9.95 (9.19–10.76)	1.58 (1.44–1.74)
Parents smoke						
No	1	1	1	1	1	1
Only one	1.59 (1.54–1.65)	1.24 (1.19–1.29)	1.84 (1.69–2.00)	1.38 (1.25–1.53)	1.63 (1.58–1.69)	1.26 (1.21–1.30)
Both smoke	1.96 (1.2–2.12)	1.29 (1.18–1.41)	2.31 (1.86–2.86)	1.39 (1.08–1.80)	2.01 (1.87–2.16)	1.30 (1.19–1.41)
Contextual						
School type						
Public	—	—	—	—	1	1
Private	—	—	—	—	0.84 (0.79–0.88)	1.01 (0.95–1.07)
Committee that coordinates health actions at school						
No	1	1	1	1	1	1
Yes	1.01 (0.96–1.06)	1.00 (0.95–1.05)	0.96 (0.88–1.06)	0.97 (0.98–1.06)	1.00 (0.95–1.04)	0.99 (0.95–1.03)
Joint actions with PC^a^ at school						
No	1	1	1	1	1	1
Yes	0.96 (0.91–1.02)	1.00 (0.95–1.06)	1.06 (0.96–1.16)	1.03 (0.94–1.12)	1.04 (0.99–1.08)	1.01 (0.97–1.06)
Implements PSE actions^b^						
No	1	1	—	—	—	—
Yes	0.93 (0.89–0.98)	0.99 (0.94–1.04)	—	—	—	—
Principal’s report of use of cigarettes by teachers in the school						
No	1	1	1	1	1	1
Yes	1.17 (1.09–1.26)	1.17 (1.10–1.26)	1.09 (0.92–1.29)	0.91 (0.75–1.11)	1.18 (1.10–1.26)	1.15 (1.08–1.23)
Principal’s report of use of cigarettes by students at school						
No	1	1	1	1	1	1
Yes	1.05 (0.99–1.11)	0.92 (0.87–0.97)	1.19 (0.98–1.45)	1.16 (0.93–1.44)	1.09 (1.03–1.15)	0.93 (0.95–1.03)
Policy banning alcohol consumption at school						
No	1	1	1	1	1	1
Yes	1.21 (1.12–1.30)	1.13 (1.05–1.21)	0.98 (0.82–1.17)	0.98 (0.82–1.18)	1.16 (1.08–1.24)	1.11 (1.04–1.18)

a PC, primary care.

b PSE, health at school program.

For the adjustment, the following variables were used: sex, age, maternal education, skin color, score of goods, information on cigarette and illicit drugs consumption by the adolescent.

OR, odds ratio; CI, confidence interval.

Being a female, having previous cigarette and drug use, having friends who use alcohol and drugs, and having smoking parents were positively associated with alcohol use in the last 30 days (Table 3). In the total model, adolescents from schools that were aware of student cigarette use were less likely to use alcohol in the last 30 days, whereas those from schools that knew about teacher cigarette use on their premises were more likely to use alcohol, both losing association in the stratified adjusted model for private schools. Although PSE actions implemented in public schools were associated with less alcohol use in the last 30 days in the crude model, the association did not hold after adjustments (Table 3).

**TABLE 3:** Odds ratio estimated by multilevel analysis for the association between individual and contextual characteristics and alcohol consumption in the last 30 days by Brazilian adolescents. National School Health Survey, Brazil, 2015.

Variables	Public schools		Private schools		Total	
	Crude model OR (95%CI)	Adjusted model OR (95%CI)	Crude model OR (95%CI)	Adjusted model OR (95%CI)	Crude model OR (95%CI)	Adjusted model OR (95%CI)
Individuals						
Sex						
Male	1	1	1	1	1	1
Female	1.11 (1.08–1.15)	1.10 (1.06–1.15)	1.11 (1.04–1.19)	1.04 (0.96–1.13)	1.11 (1.08–1.15)	1.09 (1.05–1.13)
Used cigarette in the last 30 days						
No	1	1	1	1	1	1
Yes	14.69 (13.65–15.81)	5.79 (5.33–6.30)	25.13 (20.27–31.15)	5.53 (4.31–7.09)	15.76 (14.70–16.89)	5.75 (5.32–6.23)
Used drugs in the past 30 days						
No	1	1	1	1	1	1
Yes	16.49 (15.05–18.06)	3.88 (3.49–4.31)	30.10 (23.76–38.13)	6.19 (4.71–8.14)	18.11 (16.64–19.71)	4.15 (3.76–4.58)
Having friends						
None	1	1	1	1	1	1
1 or 2 friends	1.18 (1.08–1.30)	1.11 (1.00–1.24)	1.40 (1.09–1.79)	1.45 (1.09–1.95)	1.19 (1.10–1.30)	1.15 (1.04–1.27)
3 or more friends	1.05 (0.97–1.14)	1.07 (0.97–1.18)	137 (1.08–1.73)	1.53 (1.16–2.02)	1.08 (1.00–1.17)	1.12 (1.02–1.22)
Friends use alcohol						
No	1	1	1	1	1	1
A few	6.55 (6.02–7.12)	4.78 (4.38–5.22)	7.19 (5.98–8.63)	4.93 (4.08–5.97)	6.67 (6.18–7.20)	4.79 (4.42–5.18)
Most	25.57 (23.45–27.87)	13.15 (11.98–14.44)	35.37 (29.27–42.74)	14.95 (12.19–18.32)	27.22 (25.16–29.46)	13.43 (12.34–14.62)
Friends use drugs						
No	1	1	1	1	1	1
A few	4.17 (3.99–4.35)	1.80 (1.72–1.89)	5.71 (5.24–6.23)	2.35 (2.13–2.59)	4.45 (4.28–4.62)	1.90 (1.82–1.98)
Most	9.59 (8.94–10.28)	1.60 (1.47–1.74)	17.58 (14.61–21.14)	2.34 (1.86–2.94)	10.51 (9.85–11.21)	1.68 (1.55–1.83)
Parents smoke						
No	1	1	1	1	1	1
Only one	1.57 (1.50–1.63)	1.24 (1.19–1.30)	2.20 (1.76–2.73)	1.46 (1.31–1.63)	1.62 (1.56–1.68)	1.27 (1.22–1.33)
Both smoke	2.08 (1.93–2.25)	1.42 (1.30–1.56)	2.54 (1.92–3.37)	1.30 (1.00–1.69)	2.12 (1.97–2.28)	1.41 (1.30–1.54)
Contextual						
School type						
Public		—	—	—	1	1
Private		—	—	—	0.82 (0.77–0.87)	0.91 (0.85–0.98)
Committee that coordinates health actions at school						
No	1	1	1	1	1	1
Yes	0.98 (0.93–1.04)	0.96 (0.90–1.01)	1.01 (0.89–1.14)	1.00 (0.89–1.12)	0.98 (0.93–1.03)	0.96 (0.92–1.01)
Joint actions with PC^a^ at school						
No	1	1	1	1	1	1
Yes	1.00 (0.94–1.06)	1.6 (0.9–1.13)	1.7 (0.95–1.21)	1.06 (0.95–1.19)	1.07 (1.01–1.13)	1.06 (1.00–1.12)
Implements PSE actions^b^						
No	1	1	—	—	—	—
Yes	0.93 (0.88–0.98)	0.99 (0.93–1.05)	—	—	—	—
Principal’s report of use of cigarettes by teachers in the school						
No	1	1	1	1	1	1
Yes	1.18 (1.09–1.27)	1.17 (1.09–1.26)	1.15 (0.92–1.43)	0.97 (0.76–1.24)	1.19 (1.11–1.28)	1.16 (1.08–1.24)
Principal’s report of use of cigarettes by students at school						
No	1	1	1	1	1	1
Yes	1.03 (0.97–1.10)	0.90 (0.85–0.96)	1.22 (0.95–1.57)	1.08 (0.82–1.42)	1.08 (1.01–1.14)	0.91 (0.86–0.97)
Policy banning alcohol consumption at school						
No	1	1	1	1	1	1
Yes	1.13 (1.04–1.23)	1.03 (0.95–1.12)	1.03 (0.81–1.30)	0.99 (0.79–1.24)	1.10 (1.01–1.19)	1.02 (0.95–1.11)

a PC: primary care.

b PSE: health at school program.

For the adjustment, the following variables were used: sex, age, maternal education, skin color, score of goods, information on cigarette and illicit drugs consumption by the adolescent.

OR: odds ratio; CI: confidence interval.

Being a female was negatively associated with drunken episodes, while previous cigarette and illicit drug consumption, alcohol and illicit drug consumption by peers, and having smoking parents were positively associated with drunken episodes in adolescents. As with the other outcomes, there was a positive association between schools that knew about teacher cigarette use on their premises and drunken episodes, in the total and stratified model for public schools. In the same way as for the other outcomes, the association of implementation of PSE actions with drunkenness did not hold after the adjustments (Table 4).

**TABLE 4:** Odds ratio estimated by multilevel analysis for the association between individual and contextual characteristics and drunkenness episode by Brazilian adolescents. National School Health Survey, Brazil, 2015.

Variables	Public schools		Private schools		Total	
	Crude model OR (95%CI)	Adjusted model OR (95%CI)	Crude model OR (95%CI)	Adjusted model OR (95%CI)	Crude model OR (95%CI)	Adjusted model OR (95%CI)
Individuals						
Sex						
Male	1	1	1	1	1	1
Female	0.95 (0.92–0.98)	0.91 (0.87–0.95)	0.93 (0.86–1.00)	0.83 (0.76–0.91)	0.95 (0.92–0.98)	0.90 (0.87–0.93)
Used cigarette in the last 30 days						
No	1	1	1	1	1	1
Yes	11.72 (10.94–12.56)	4.01 (3.70–4.34)	21.92 (18.05–26.62)	4.20 (3.34–5.29)	12.78 (11.98–13.64)	4.03 (3.74–4.35)
Used drugs in the past 30 days						
No	1	1	1	1	1	1
Yes	16.45 (15.04–17.99)	3.68 (3.32–4.08)	30.04 (24.13–37.40)	5.43 (4.20–7.00)	18.14 (16.70–19.71)	3.93 (3.58–4.32)
Having friends						
None	1	1	1	1	1	1
1 or 2 friends	1.16 (1.06–1.27)	1.14 (1.03–1.27)	0.82 (0.65–1.03)	0.75 (0.57–0.97)	1.10 (1.01–1.20)	1.08 (0.98–1.19)
3 or more friends	0.93 (0.86–1.02)	0.96 (0.87–1.06)	0.78 (0.63–0.97)	0.76 (0.59–0.97)	0.91 (0.84–0.98)	0.93 (0.85–1.02)
Friends use alcohol						
No	1	1	1	1	1	1
A few	4.94 (4.57–5.34)	3.21 (2.96–3.48)	6.35 (5.22–7.73)	4.20 (3.42–5.16)	5.11 (4.75–5.50)	3.32 (3.07–3.58)
Most	18.25 (16.83–19.78)	7.62 (6.98–8.33)	32.50 (26.57–39.75)	12.49 (10.04–15.53)	20.9 (18.63–21.65)	8.21 (7.57–8.91)
Friends use drugs						
No	1	1	1	1	1	1
A few	4.90 (4.69–5.13)	2.39 (2.28–2.51)	6.34 (5.78–6.96)	2.61 (2.35–2.90)	5.16 (4.96–5.37)	2.44 (2.34–2.55)
Most	12.18 (11.36–13.06)	2.60 (2.39–2.83)	24.48 (20.32–29.48)	3.50 (2.79–4.39)	13.54 (12.6–14.4)	2.69 (2.49–2.91)
Parents smoke						
No	1	1	1	1	1	1
Only one	1.67 (1.60–1.74)	1.34 (1.28–1.40)	2.04 (1.85–2.25)	1.58 (1.41–1.77)	1.73 (1.67–1.80)	1.37 (1.31–1.43)
Both smoke	2.25 (2.09–2.44)	1.58 (1.45–1.73)	2.62 (2.10–3.27)	1.56 (1.20–2.04)	2.32 (2.16–2.50)	1.58 (1.45–1.72)
Contextual						
School type						
Public	—	—	—	—	1	1
Private	—	—	—	—	0.73 (0.69–0.78)	0.87 (0.82–0.93)
Committee that coordinates health actions at school						
No	1	1	1	1	1	1
Yes	1.00 (0.94–1.05)	0.98 (0.93–1.04)	0.98 (0.87–1.10)	0.99 (0.90–1.10)	0.99 (0.94–1.04)	0.98 (0.93–1.02)
Joint actions with PC^a^ at school						
No	1	1	1	1	1	1
Yes	0.98 (0.92–1.05)	1.06 (1.00–1.12)	1.5 (0.93–1.18)	1.02 (0.92–1.13)	1.08 (1.03–1.14)	1.04 (0.99–1.10)
Implements PSE actions^b^						
No	1	1	—	—	—	—
Yes	0.91 (0.86–0.96)	0.96 (0.91–1.01)	—	—	—	—
Principal’s report of use of cigarettes by teachers in the school						
No	1	1	1	1	1	1
Yes	1.17 (1.08–1.27)	1.13 (1.06–1.22)	1.19 (0.96–1.47)	1.04 (0.84–1.30)	1.20 (1.12–1.30)	1.13 (1.06–1.21)
Principal’s report of use of cigarettes by students at school						
No	1	1	1	1	1	1
Yes	1.11 (1.04–1.18)	0.97 (0.91–1.03)	1.24 (0.97–1.58)	1.06 (0.83–1.36)	1.17 (1.10–1.25)	0.97 (0.92–1.03)
Policy banning alcohol consumption at school						
No	1	1	1	1	1	1
Yes	1.16 (1.06–1.27)	1.05 (0.97–1.13)	0.94 (0.74–1.18)	0.91 (0.73–1.12)	1.10 (1.02–1.20)	1.03 (0.95–1.11)

a PC, primary care.

b PSE, health at school program.

For the adjustment, the following variables were used: sex, age, maternal education, skin color, score of goods, information on cigarette and illicit drugs consumption by the adolescent.

OR, odds ratio; CI, Confidence Interval.

## Discussion

This study, which analyzed data from more than one hundred thousand Brazilian adolescents, found that girls had a greater chance of alcohol experimentation and consumption in the last 30 days, although boys were more likely to get drunk. There was a positive association between substance use by parents, peers and the adolescent himself and all outcomes of alcohol consumption by them. Students whose schools had a policy banning alcohol were more likely to try alcoholic drinks and students whose principals reported that teachers smoked on school premises had greater alcohol use in all of their outcomes in the set of schools and in public schools.

The greater alcohol experimentation and consumption in the last 30 days by girls was observed in previous surveys [3, 24]. In contrast, boys were more likely to get drunk, a fact that can be justified by the standards and customs present in society, where excessive alcohol consumption by females is still frowned upon [25]. Moreover, males tend to adopt more risky behaviors, considering heavy drinking as a challenging attitude and maintaining their consumption in an abusive and problematic way [24–27].

Adolescents who reported alcohol use by friends had a positive association for alcohol consumption in the three different outcomes, corroborating previous reports in the literature [28]. This finding can be explained by the fact that peers are identified as the main alcohol suppliers for adolescents [29], and because drinking is considered a behavior of peer acceptance [20, 30]. In contrast, adolescents tend to approach people who have similar habits and customs, so students who have consumed alcohol would have friends who share the same lifestyle [31].

Adolescents who had a smoking parent were more likely to use alcohol in its three outcomes, and if both parents smoked the chances were even greater. The smoking habit is commonly associated with drinking, and therefore, a family environment permissive about cigarette use may also reflect greater tolerance to alcohol [24]. Parents and friends are a source of support, protection and an example to adolescents and, therefore, their attitudes can be replicated by young people [32].

Previous consumption of cigarettes and illicit drugs by students was associated with the three outcomes of alcohol use. This fact can be explained by the multiplier effect caused by substance use, where in most cases alcohol and cigarettes are considered as a gateway to illicit drug use [9, 24, 33]. Although alcohol consumption is associated with other substance consumption, the present study did not analyze which substance was the initiation one.

The existence of an alcohol ban policy had a positive association with alcohol experimentation in public schools. It is possible that schools have created policies to ban alcohol consumption after detecting the growing use of alcohol by adolescents, and for this reason our data show worse results of experimentation in schools with such policies. In addition, this study failed to identify which policies were adopted by schools, making it impossible to differentiate between stricter and more lenient measures. Differently from our results, a study carried out in the United States and Australia found that low enforcement of alcohol ban policies at school increased the chances of both use in the last 30 days and episode of drunkenness by adolescents [34]. Alcohol ban policies at school may have different effects than preventive policies. The problem, according to Beauchesne [35] and Veríssimo [36], is that very restrictive prohibitionist policies tend to decrease the production of knowledge about substances, such as alcohol and drugs, and the consequences of their use, directly impacting the adolescent’s ability to deal with the use of these substances.

Positive effects on the creation of alcohol consumption prevention programs or policies have been shown to be effective in some studies. European studies have identified a lower propensity in the progress of frequent alcohol consumption and lower chances of alcohol-related problems, in addition to a reduction of 29% in the chances of consuming alcoholic drinks and of 43% in abusive drinking [37]. In contrast, other studies have found no association between preventive policies and alcohol consumption reduction, delayed experimentation and episode of drunkenness [18, 38, 39]. It is worth mentioning that some interventions fail because they do not take into account individual, contextual and type of consumption factors and there is no conclusive information in the literature regarding why alcohol use prevention programs are beneficial for some groups of adolescents and not for others [19, 37, 39].

The development of joint health actions with PC was not associated with alcohol use in any of the outcomes, but there was a higher prevalence of these actions in public than in private schools. PC should be the point of reference for the entire community, including schools, in health promotion and disease prevention actions, due to the presence of professionals qualified to deal with the topic [40]. School curriculum with programs that encompass individual aspects, family and community involvement, teacher training and joint actions with specialists in prevention campaigns are more effective in reducing adolescent substance use [10, 41].

PSE was evaluated only in the stratified models for public schools, since in Brazil this policy is exclusive to the public sector [14, 40]. Students of schools that implemented PSE actions were less likely to use alcohol in the three outcomes of unadjusted models. In this context, it is possible that alcohol use was related to individual characteristics rather than to contextual variables, explaining the loss of association after making the adjustments. In addition, only 43.1% of public schools implemented PSE actions. In contrast, a study that evaluated the implementation of PSE actions highlighted problems such as activities attributed to health services with limited school participation, and insufficient results in the adolescents’ attitudes [42]. It is worth mentioning that alcohol preventive actions are essential actions of the program, but their implementation is not mandatory in all schools [14, 40].

When teachers smoked on school premises and the school community was aware of this fact, students were more likely to use alcohol in the three outcomes. As the school is the teaching place, the school’s acceptance of teachers smoking at school can indicate to students that substance use is not a problem and may work as a model [35, 43].

In schools that were aware of adolescent cigarette use on their premises, students were less likely to consume alcohol in the last 30 days. A possible explanation for this finding is that the knowledge of the school management about cigarette consumption on its premises may be the result of the stricter monitoring of students by the school, potentially adopting measures to control substance use that led to the protection of young people and, thus, the lower possibility of students having used alcohol recently [44].

Adolescents in public schools consumed more substances than those in private schools. The former had a lower socioeconomic level, assessed by asset score tertiles, than the latter, which could explain the difference. The low socioeconomic status was associated with a higher incidence of alcohol abuse among adolescents in a previous study [45]. The hypothesis raised by the researchers was that low income was associated with low parental education and, therefore, the scarcity of information from the family nucleus increased the adolescents chances of consuming alcohol in an abusive way.

Our results do not corroborate the hypothesis that the different alcohol outcomes could have different predictors [21]. Most of the associations with alcohol use determinants in the three outcomes were the same. Thus, policies tackling these exposures could potentially have a positive impact in alcohol experimentation and use in the last 30 days and drunkenness episode among adolescents.

Finally, it should be noted that, in Brazil, the sale of alcoholic beverages to persons under 18 is prohibited, pursuant to article 243 of the Child and Adolescent Statute (Law 8,069/1990) and by the Criminal Misdemeanors Law, article 63 [46]. Since March 2015, selling, supplying, serving, administering or delivering alcoholic beverages to a child or adolescent, even if free of charge, is liable to imprisonment for two to 4 years and a fine. In addition, advertising is restricted to beverages with an alcohol content equal to or greater than 0.5-degree Gay Lussac, and advertisements can only be aired on radio and television stations between 9 pm and 6 am [47]. In the long term, better results are expected with the implementation and enforcement of these measures.

Despite this, studies have shown the consumption of alcoholic beverages by an important portion of adolescents in the country [47–50]. In addition to the factors already discussed, the lack of enforcement of laws, low price of alcoholic beverages, few restrictions on alcohol advertising in the media, wide availability of alcohol in environments, bars operating under a minimum consumption system and “open bar” promotions increase the risk of problems related to alcohol consumption in this age group [51].

Therefore, the findings of this study highlight the need to strengthen preventive and restrictive programs for alcohol use in schools, in addition to other policies that regulate the alcohol market (such as tax increases) and prevent early consumption of alcoholic beverages, always accompanied by continuous evaluations of the effectiveness of these measures.

### Strengths and Limitations

This study’s strengths include sample size, high response rate (98.6%), the representativeness for entire Brazilian territory, and possibility of determining if the existence of a committee that coordinates health activities at school, if carrying out joint actions with PC, and if implementing PSE actions at school were associated with adolescent alcohol consumption. It is also worth mentioning that multilevel analysis allows analyzing the effect of contextual variables taking into account the possible correlation between data from the same group. However, some limitations must be considered. First, it is a cross-sectional study, making it impossible to establish a causal relationship. Second, the possibility of omitted variables, measurement errors of the variables included in the model and/or simultaneity between dependent and independent variables, generating some endogeneity problem. Third, self-reported information allows for underestimation or overestimation of the indicators studied, depending on the lower or higher social acceptance of the evaluated behaviors, the difficulty in understanding the questions or even the memory bias. In addition, the PeNSE [22, 23] questions did not allow to identify what the alcohol ban policy on school premises was like and which PC actions were developed at school. Furthermore, it was not possible to identify whether the PSE actions involved the theme of alcohol, limiting our ability to differentiate levels of implementation of these actions and policies. Another point is the fact that the survey was conducted only with adolescents enrolled and present on the day the questionnaire was applied, excluding absent students or dropouts. The literature shows that those most exposed to psychotropic substance use are also more prone to school absenteeism and dropout [3]. Regarding the multilevel analysis, it was not possible to consider the complex sampling design in the models due to operational limitations of the software. However, previous studies have found no differences in the results of association analysis when comparing models that accounted for the sample design with models that did not [52, 53]. Despite this, the standard errors and consequently the 95% CI might have been affected by not incorporating the complex sample design in our analysis; therefore, the readers should interpret any weak associations with caution.
